# Protein expression of the amino acid transporter SLC7A5 in tumor tissue is prognostic in early-stage colorectal cancer

**DOI:** 10.1371/journal.pone.0298362

**Published:** 2024-05-09

**Authors:** Makiko Ogawa, Atsushi Tanaka, Masaki Maekawa, Kei Namba, Yusuke Otani, Jinru Shia, Julia Y. Wang, Michael H. Roehrl

**Affiliations:** 1 Department of Pathology, Beth Israel Deaconess Medical Center, Harvard Medical School, Boston, MA, United States of America; 2 Harvard Medical School, Boston, MA, United States of America; 3 Department of Pathology and Laboratory Medicine, Memorial Sloan Kettering Cancer Center, New York, NY, United States of America; 4 Curandis, New York, NY, United States of America; Qatar Biomedical Research Institute, QATAR

## Abstract

Proteins overexpressed in early-stage cancers may serve as early diagnosis and prognosis markers as well as targets for cancer therapies. In this study, we examined the expression of an essential amino acid carrier SLC7A5 (LAT1, CD98, or 4F2 light chain) in cancer tissue from two well-annotated cohorts of 575 cases of early-stage and 106 cases of late-stage colorectal cancer patients. Immunohistochemistry showed SLC7A5 overexpression in 72.0% of early-stage and 56.6% of late-stage cases. SLC7A5 expression was not influenced by patient gender, age, location, or mismatch repair status, although it appeared to be slightly less prevalent in tumors of mucinous differentiation or with lymphovascular invasion. Statistical analyses revealed a positive correlation between SLC7A5 overexpression and both overall survival and disease-free survival in early-stage but not late-stage cancers. Co-expression analyses of the TCGA and CPTAC colorectal cancer cohorts identified a network of gene transcripts positively related to SLC7A5, with its heterodimer partner SLC3A2 having the highest co-expression score. Network analysis uncovered the SLC7A network to be significantly associated with ncRNA such as tRNA processing and the mitotic cell cycle. Since SLC7A5 is also a marker of activated lymphocytes such as NK, T, and B lymphocytes, SLC7A5 overexpression in early colorectal cancers might trigger a strong anti-tumor immune response which could results in better clinical outcome. Overall, our study provides clear evidence of differential SLC7A5 expression and its prognostic value for early-stage colorectal cancer, although the understanding of its functions in colorectal tumorigenesis and cancer immunity is currently rather limited and awaits further characterization.

## Introduction

Colorectal cancer is one of the most common and lethal malignancies worldwide. Despite enormous advances in cancer treatments, early detection and risk stratification remain a key for optimal clinical outcome in patient care. Early diagnosis and prognosis are currently hindered by insufficient predictable biomarkers in early-stage cancers. Given that cancer therapies are generally targeting protein biomarkers and their function pathways, we sought to discover proteins that emerge early in cancer development and exert critical functions in tumorigenesis. In this study, we investigated the expression of an essential amino acid transporter SLC7A5 (solute carrier family 7 member 5) in colorectal cancer tissue and for its prognostic potential in early-stage disease.

SLC7A5 has several aliases, including LAT1 (L-type amino acid transporter 1), CD98 light chain, 4F2 light chain, large neutral amino acids transporter 1, and integral membrane protein E16 [1–3]. SLC7A5 functions as a membrane transporter and facilitates the cross-membrane flux of large neutral amino acids, e.g., leucine, methionine, phenylalanine, tyrosine, histidine, tryptophane [1, 4, 5]. LAT1 mRNA becomes expressed in various tumor types with high frequency, but expression appears restricted to normal cells of certain organs such as the brain, spleen, placenta, and bone marrow [5, 6]. SLC7A5 also mediates the transport of thyroid hormones and hormone precursors across the cell membrane, L-DOPA across the blood-brain barrier, L-leucine across the blood-retinal barrier, amino acid-polyamine-organocation superfamily pharmaceutical drugs, toxic methylmercury, 3-(18)F-l-α-methyl-tyrosine PET probe in cancer imaging, and others [7–11].

Functional studies have shown that SLC7A5 plays various important roles in tumorigenesis. In human cancer cell lines from colon, lung and kidney, SLC7A5 transport activity was the key growth-limiting step [12]. In melanoma cells, enhanced SLC7A5 expression increased the uptake of essential amino acids and the subsequent maintenance of mTORC1 activity, and blocking the MAPK-c-MYC-SLC7A5 signaling axis effectively suppressed cancer cell growth [13]. In mouse models, SLC7A5 was upregulated in oncogenic KRAS-mutant colorectal cancer cells [14]; SLC7A5 helped maintain intracellular amino acid levels and supported the increased protein synthesis that underpins the enhanced KRAS-mutant cell proliferation, whereas targeting protein synthesis via mTOR regulator and SLC7A5 abrogated the growth of established KRAS-mutant tumors [15].

SLC7A5 has been reported to be prognostic in several types of cancer, including breast cancer [16–18], pancreatic cancer [19], tongue cancer [20], renal cell carcinoma [21], and esophageal squamous cell carcinoma [22]. Among early-stage cancers, SLC7A5 has been reported to be prognostic in stage I squamous cell carcinoma of the lung [23] and pulmonary adenocarcinoma [24]. In colorectal cancer, immunohistochemical staining of 174 cancer tissues suggested SLC7A5 to be a predictive marker [2], although another study of a mixed cohort of 351 colorectal cancer patients did not find similar prognostic significance [25]. In a third cohort of 210 colorectal cancer cases, SLC7A5 high expression was observed in 72.4% of the patients but no prognostic conclusion was given [26]. To clarify and define the role of SLC7A5 expression in colorectal cancer, we investigated two well-annotated cohorts of 681 colorectal cancer patients, including 575 early-stage and 106 late-stage cancers, as well as the TCGA and CPTA colorectal cancer cohorts for complimentary insights.

## Materials and methods

### Clinical case selection and pathological data

Colorectal cancer tissue specimens from 681 patients were obtained from the Precision Pathology Biobank of Memorial Sloan Kettering Cancer Center (MSKCC). The cohorts comprise of 575 cases of early-stage (AJCC stages I or II) and 106 cases of late-stage (AJCC stages III or IV) colorectal cancer. These tumor tissues had been surgically resected at MSKCC. The study was approved by MSKCC’s Institutional Review Board, and clinical data and archival specimens were acquired retrospectively in an anonymized manner such that the need for informed consent was waived. Clinical parameters, including patient age, treatment history, recurrence, and survival status, were retrieved from medical records. Histologic type and other clinicopathological parameters of all samples were verified by pathologists with gastrointestinal subspecialty.

### Tissue microarray (TMA) construction

Tissue microarrays were constructed from the 681 colorectal tumors. All archival tissue specimens had been fixed with formalin and embedded in paraffin blocks. Three 0.6-mm tissue cores were drilled out from each donor tissue block and transferred to tissue array blocks using a TMA Grand Master robot (3DHistech). The cored areas were defined by a certified pathologist for each case, and the tissue cores included tumor tissue as well as paired normal mucosal tissue.

### Immunohistochemistry (IHC)

The tissue microarray blocks were cut into 4-μm sections. Paraffin was removed with xylene, and antigens were retrieved by a BOND epitope retrieval solution 2 (EDTA buffer, pH 9.0) performed on the Leica BOND RX slide stainer for 40 min at 100°C. Tissue sections were incubated with SLC7A5-specific polyclonal antibodies (HPA052673, 1:50, Atlas Antibodies, Sigma) for 30 min. They were followed by visualization with the Bond Polymer Refine Detection (DS9800, Leica).

### Immunohistochemical scoring

IHC stained tissue slides were evaluated independently by two pathologists without knowledge of the patients’ clinical information. The total staining intensity of tumor cells was determined and assigned values of 0, 1, 2, or 3, corresponding to negative, weak, medium, and strong staining, respectively. For each slide, an IHC H-score (the total weighted IHC score) was calculated by multiplying the expression intensity of individual tumor areas (score, 0–3) by their relative distribution (0–100%) to total tumor area and adding these to yield a total weighted sum. IHC H-scores therefore have a theoretical range of 0–300. The average score across 3 independent tissue cores was calculated for each case. Based on H-score distribution, a tissue sample was considered having high- or overexpression of SLC7A5 when its H-score was ≥40, otherwise it was considered low expression.

### cBioPortal dataset analysis

Two colorectal cancer cohorts, including a 594-cases cohort from the Cancer Genome Atlas (TCGA, PanCancer Atlas) [27, 28], and a 110-case cohort from the Clinical Proteomics Tumor Assessment Consortium (CPTAC) [29] were analyzed for gene and protein expression levels of SLC7A5, KRAS, BRAF, PIK3CA, and all others in the database for possible co-expression. All these sequencing results and relevant clinical information were downloaded from cBioPortal.

### SLC7A5 co-expression network analysis

Functional enrichment analysis of the SLC7A5-coexpression network was performed using STRING [30]. The analysis was based on a database of known and predicted protein-protein interactions, which include direct physical interactions and indirect functional associations. These interactions stem from genomic context prediction, lab experiments, knowledge transformation between organisms, automated text mining, and previous knowledge in databases. Interactions were scored between 0 and 1, with 1 being the highest possible confidence. In this study, only those protein-protein interactions with confidence scores >0.7 (high confidence) were considered.

### Statistical analysis

Categorical variables were compared using Fisher’s exact test. Survival time analyses were conducted with the Kaplan-Meier method and compared by a log-rank test. To select initial candidate factors for multivariate analyses, we performed univariate logistic regression analyses. Then, we included individual factors with statistical significance (p<0.05) in a multivariate logistic regression model. To select final factors for a final model, we removed factors with highest p-values one by one until all remaining factors were statistically significant (i.e., p<0.05), using a backward elimination method [31]. Correlation coefficients were calculated with the Spearman method. Statistical analyses were performed by JMP Pro 14 software (SAS).

## Results and discussion

### SLC7A5 protein expression in normal colon mucosa and colorectal cancer

**Fig 1:**
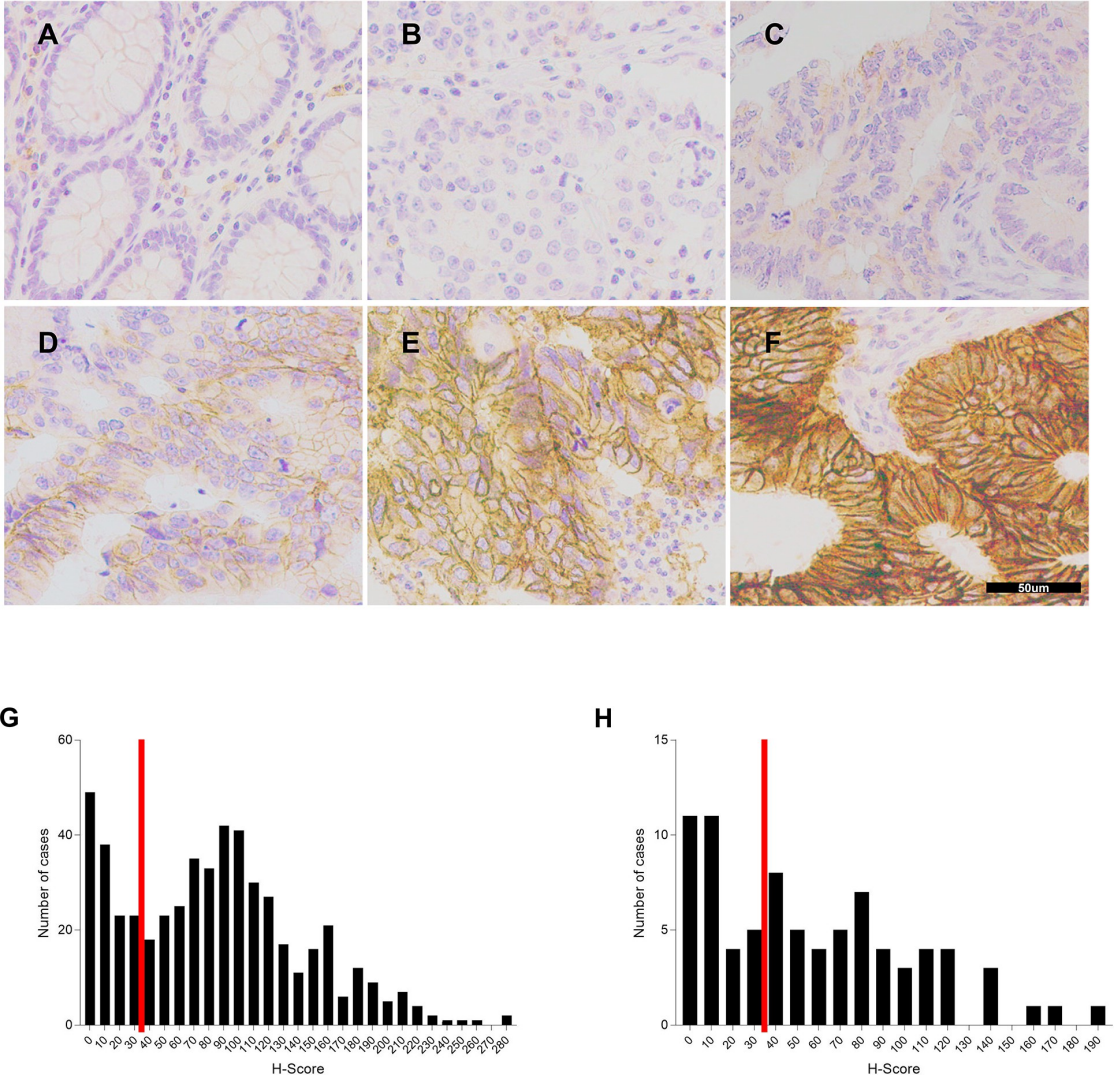
SLC7A5 protein expression patterns and range in colorectal cancer tissues and normal mucosa. SLC7A5 proteins are stained in brown; cell nuclei are counter stained purple. Original magnifications: 400×. (**A**) In benign mucosa, SLC7A5 expression is negative to very weak and localized to the cytoplasmic membrane or stromal cytoplasm. (**B-F**) In colorectal cancer, SLC7A5 is detected in tumor membrane at various levels, ranging from weakly positive tumor cells to strong expression in most cells. (**G, H**) SCL7A5 expression range distribution in 522 earl-stage cancers (**G**) and 81 late-stage cancers (**H**). Total weighted H-score of 40 is used as a cut-off (vertical red line). SLC7A5 expression in **B, C** are considered low and in **D-F** are considered high.

We performed immunostaining of normal colonic and rectal mucosa to investigate the expression of SLC7A5 protein in normal intestinal tissue. In normal colorectal mucosa, SLC7A5 protein expression was barely detectable and localized to the cytoplasmic membrane if detected (Fig 1A). Similar to their benign enterocyte counterparts, stromal cells express none to very low levels of SLC7A5. In contrast, colorectal cancers in different patients exhibited a wide range of SLC7A5 expression in tumor membrane, with weakly positive in some tumor cells to strongly positive in most tumor cells (Fig 1B–1F).

To better comprehend the differential protein expression of SLC7A5 in colorectal cancer, we immunohistochemically stained cancer tissues from 681 patients with stages I-IV colorectal cancer that had not received neoadjuvant therapy. We calculated the total stanning intensities (H-score) for each cancer tissue. For both early-stage (stage I and II) and late-stage (stage III and IV) cancers, SLC7A5 expression showed a broad distribution range across our patient cohorts (Fig 1G and 1H). Based on the expression distribution histograms, we separated tumors into two groups using H-score of 40 as a cut off. Cancer tissues with H-scores of over 40 were considered having SLC7A5 over or high expression (Fig 1G and 1H). The expression ranges and patterns of early-stage and late-stage cohorts were similar. In the 575-case early-stage cancer cohort, 72.0% of the cases had high expression and 28.0% had low expression. In the 106-case late-stage cancer cohort, 56.6% had high expression and 43.4% had low expression.

### SLC7A5 protein expression vs. clinicopathological features

To examine the correlation of SLC7A5 protein expression with clinicopathological features of the cancer patients, we analyzed various parameters, including gender, age, tumor histology, tumor differentiation, and others (Table 1). In both the early-stage and late-stage cancer cohorts, high vs. low SLC7A5 expression levels showed no significant associations with the following parameters: patient gender, age, tumor differentiation (G1/G2 vs. G3), tumor location (left- vs. right-sided), perineural invasion status, or mismatch repair status (MMR) MSS vs. MSI subtype.

**Table 1:** Correlation of SLC7A5 protein expression with clinicopathological features of colorectal cancer patients.

	Early stage (n = 575)			Late stage (n = 106)		
	SLC7A5 H-score		p-value*	SCL7A5 H-score		p-value
	High	Low		High	Low	
**Total**	414 (72.0%)	161 (28.0%)		60 (56.6%)	46 (43.4%)	
**Gender**			0.3065			0.3275
Male	210 (70.0%)	90 (30.0%)		26 (51.0%)	25 (49.0%)	
Female	204 (74.2%)	71 (25.8%)		34 (61.8%)	21 (38.2%)	
**Age (years)**			1.0000			0.3996
≤70	241 (71.9%)	94 (28.1%)		58 (58.0%)	42 (42.0%)	
>70	173 (72.1%)	67 (27.9%)		2 (33.3%)	4 (66.7%)	
**Histology**			**0.0235**			0.0538
Not mucinous	389 (73.3%)	142 (26.7%)		57 (60.0%)	38 (40.0%)	
Mucinous	25 (56.8%)	19 (43.2%)		3 (27.3%)	8 (72.7%)	
**Tumor differentiation**			0.8695			0.4158
G1/G2	378 (71.9%)	148 (28.1%)		53 (58.2%)	38 (41.8%)	
G3	36 (73.5%)	13 (26.5%)		7 (46.7%)	8 (72.7%)	
**Location**			0.1372			0.1521
Left	194 (69.0%)	87 (31.0%)		36 (51.4%)	34 (48.6%)	
Right	220 (74.8%)	74 (25.2%)		24 (66.7%)	12 (33.3%)	
**Lymphovascular invasion**			**0.0424**			0.2507
Absent	365 (73.6%)	131 (26.4%)		15 (46.9%)	17 (53.1%)	
Present	49 (62.0%)	30 (38.0%)		45 (60.8%)	29 (39.2%)	
**Perineural invasion**			0.2157			0.2291
Absent	395 (72.6%)	149 (27.4%)		41 (61.2%)	26 (38.8%)	
Present	19 (61.3%)	12 (38.7%)		19 (48.7%)	20 (51.3%)	
**TNM stage**			**0.0427**			
I	162 (77.1%)	48 (22.9%)				
II	252 (69.0%)	113 (31.0%)				
						0.5179
III				42 (54.5%)	35 (45.5%)	
IV				18 (62.1%	11 (37.9%)	
**MMR**			1.0000			0.7733
Intact (MSS)	318 (72.0%)	124 (28.1%)		53 (57.6%)	39 (42.4%)	
Lost (MSI)	96 (72.2%)	37 (27.8%)		7 (50.0%)	7 (50.0%)	

In early-stage cancers, SLC7A5 overexpression was independent of patient age group at diagnosis, occurring in 71.9% of younger patients (≤70 years) vs. 72.1% of older patients (>70 years). In late-stage cancers, SLC7A5 overexpression appeared to occur more frequently in younger patients (58.0%) than in older patients (33.3%), although the difference was not statistically significant (p = 0.3996). In the early-stage cohort, SLC7A5 expression appeared to correlate with cancer stage, with overexpression found in 162/210 (77.1%) of stage I and 252/365 (69%) of stage II patients (p = 0.0427). In the late-stage cohort, SLC7A5 high expression was found in 42/77 (55.5%) of stage III and 18/29 (62.1%) of stage IV patients with no significant difference.

In the early-stage cohort, SLC7A5 expression showed statistically significant correlation with cancer histology and lymphovascular invasion status (Table 1). In these early-stage tumors, SLC7A5 overexpression was found in 389/531 (73.3%) non-mucinous and 25/44 (56.8%) mucinous histology tumors (p = 0.0235). In addition, SLC7A5 high expression was found in 365/496 (73.6%) lymphovascular invasion absent tumors and 49/79 (62.0%) invasion present tumors (p = 0.0424) in the early-stage cohort. In the late-stage cohort, similar significant differences were not found.

### SLC7A5 protein expression vs. survival time in colorectal cancer patients

**Fig 2:**
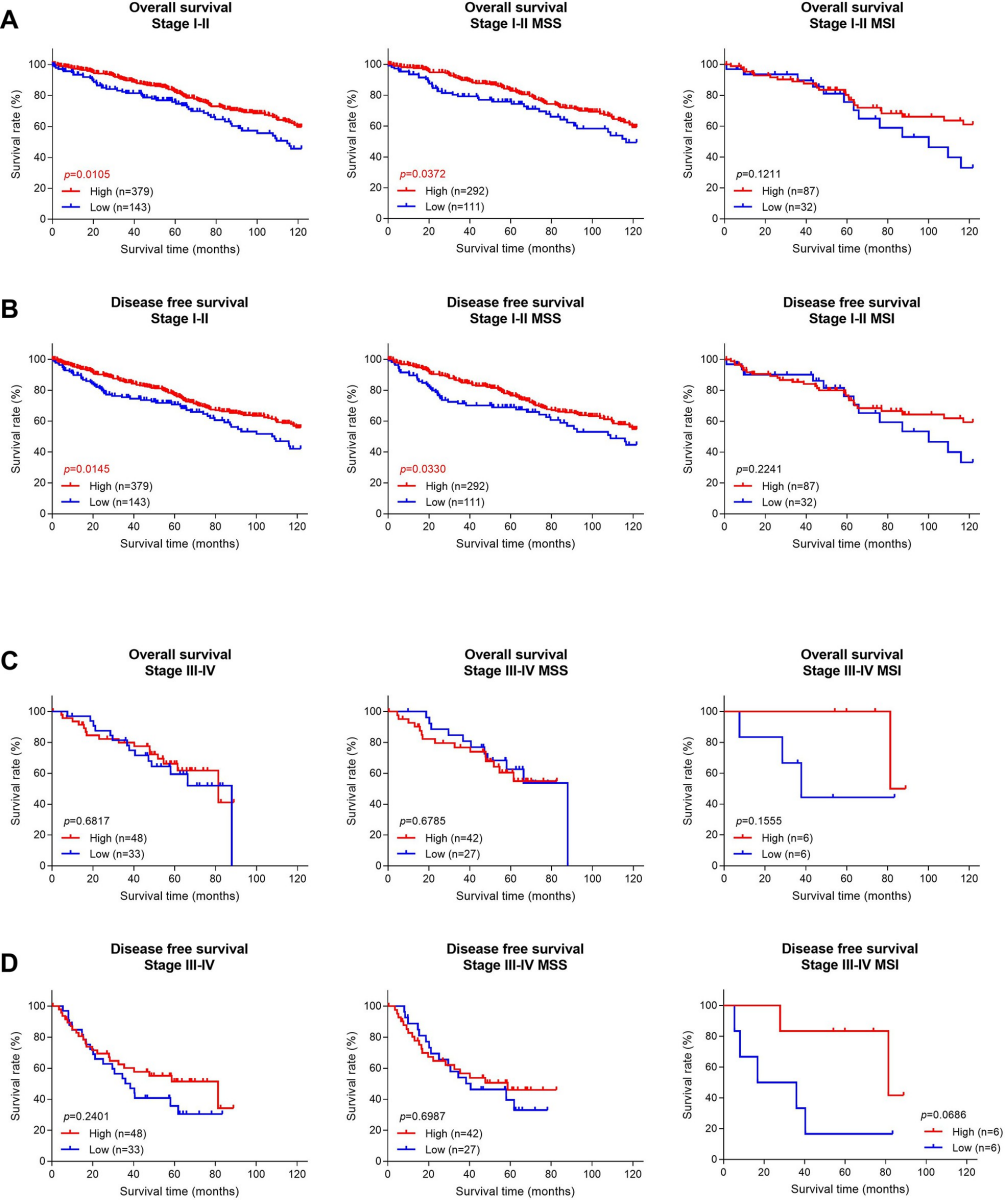
Overall survival (OS) or disease-free survival (DFS) in colorectal cancer patients stratified by SLC7A5 protein expression. High expression refers to cancer tissue with immunohistochemical staining H-score ≥40. (**A, B**) In early-stage cancers, SLC7A5 high expression is significantly correlated with longer survivals and better clinical outcome. Similar significant correlations hold in the MSS (microsatellite stability) and MSI (microsatellite instability) subtypes. (**C, D**) In late-stage cancers, SLC7A5 expression is not correlated with overall or disease-free survivals.

To find out whether SLC7A5 expression in colorectal cancer tissue is correlated with patient survival, we performed Kaplan-Meier analyses of 522 early-stage and 81 late-stage patients whose survival data was available (Fig 2). Both the overall survival time and disease-free survival time were analyzed. The stage I and II patients of this study had been followed for a range of 0.2 to 120 months, with a mean follow-up time of 70.7 months and a median follow-up time of 68.4 months. The stage III and IV patients of this study had been followed for a range of 0.4 to 88.8 months, with a mean follow-up time of 48.5 months and a median follow-up time of 53.3 months.

In early-stage colorectal cancers, SLC7A5 high expression was significantly associated with both longer overall survival and longer disease-free survival (p = 0.0105 and p = 0.0145, respectively) among the 522 patients (Fig 2). Particularly, the correlation between SLC7A5 high expression and longer survival was the most pronounced in stage II patients (p = 0.0004 for overall survival and p = 0.0005 for disease-free survival) (S1 Fig). Moreover, among the stage II cases, the MSS subtype cancer showed positive correlation between high SLC7A5 levels with both overall and disease-free survival, whereas the MSI subtype showed significant correlation with overall survival but not disease-free survival (S1 Fig).

In the late-stage colorectal cancer cohort, SLC7A5 expression levels showed no significant correlation with either overall survival or disease-free survival (Fig 2). Separate examination of patients with MSS or MSI subtype cancer also showed no significant correlation.

To further verify whether SLC7A5 is an independent prognostic factor in early-stage colorectal cancer, we performed both univariate and multivariate analyses (Table 2). Univariate analysis revealed that SLC7A5 protein high expression was an independent indicator for a better outcome in overall and disease-free survival. Moreover, in stage II patients, both univariate and multivariate analyses showed that SLC7A5 high expression was an independent indicator for longer overall and disease-free survival. Univariate analysis also revealed that SLC7A5 expression was an independent predictor for overall and disease-free survival for patients with the MSS subtype of early-stage colorectal cancer (S1 Table).

**Table 2:** Univariate and multivariate analyses of patient survival in early-stage colorectal cancer.

	Overall survival				Disease-free survival			
Variables	Univariate		Multivariate		Univariate		Multivariate	
	HR (95% CI)	p-value	HR (95% CI)	p-value	HR (95% CI)	p-value	HR (95% CI)	p-value
**Gender**	1.02	0.7569			0.95	0.4730		
(male vs. female)	(0.88–1.20)				(0.82–1.10)			
**Age (years)**	0.63	**<0.0001**	0.63	**<0.0001**	0.69	**<0.0001**	0.69	**<0.0001**
(>70 vs. ≤70)	(0.53–0.74)		(0.53–0.74)		(0.59–0.80)		(0.60–0.80)	
**Tumor location**	0.86	0.0570			0.92	0.2700		
(right vs. left)	(0.73–1.00)				(0.79–1.07)			
**Histology**	1.19	0.2821			1.13	0.4113		
(mucinous vs. other)	(0.88–1.73)				(0.85–1.58)			
**Tumor differentiation**	0.97	0.8398			1.02	0.8987		
(G3 vs. G1/2)	(0.73–1.36)				(0.78–1.40)			
**Lymphovascular invasion**	0.74	**0.0071**	0.77	**0.0268**	0.70	**0.0004**	0.73	**0.0028**
	(0.61–0.92)		(0.63–0.97)		(0.58–0.84)		(0.61–0.89)	
**Perineural invasion**	0.61	**0.0032**	0.69	**0.0258**	0.64	**0.0043**	0.75	0.0613
	(0.47–0.84)		(0.52–0.95)		(0.49–0.86)		(0.57–1.01)	
**AJCC stage**	0.76	**0.0010**	0.81	**0.0183**	0.75	**0.0003**	0.80	**0.0070**
(II vs. I)	(0.63–0.90)		(0.68–0.97)		(0.63–0.88)		(0.68–0.94)	
**MMR**	0.95	0.5504			1.02	0.8664		
(lost vs. intact)	(0.79–1.14)				(0.86–1.22)			
**SLC7A5 expression**	1.24	**0.0136**	1.19	0.0528	1.21	**0.0180**	1.15	0.0936
(Low vs. High)	(1.05–1.46)		(1.00–1.40)		(1.03–1.42)		(0.98–1.34)	

In addition, patient age and lymphovascular or perineural invasion were factors differentiating patient overall and disease-free survival. Other clinical features, such as gender, tumor location, histology, tumor grade, and mismatch repair (MMR) status, did not significantly influence clinical overall survival or disease-free survival in a multivariate model. Overall, the above statistical analyses support SLC7A5 protein expression in cancer tissue as an independent predictor for patient survival in early-stage colorectal cancer.

### SLC7A5 vs. *BRAF*, *KRAS*, and *PIK3CA* status

**Fig 3:**
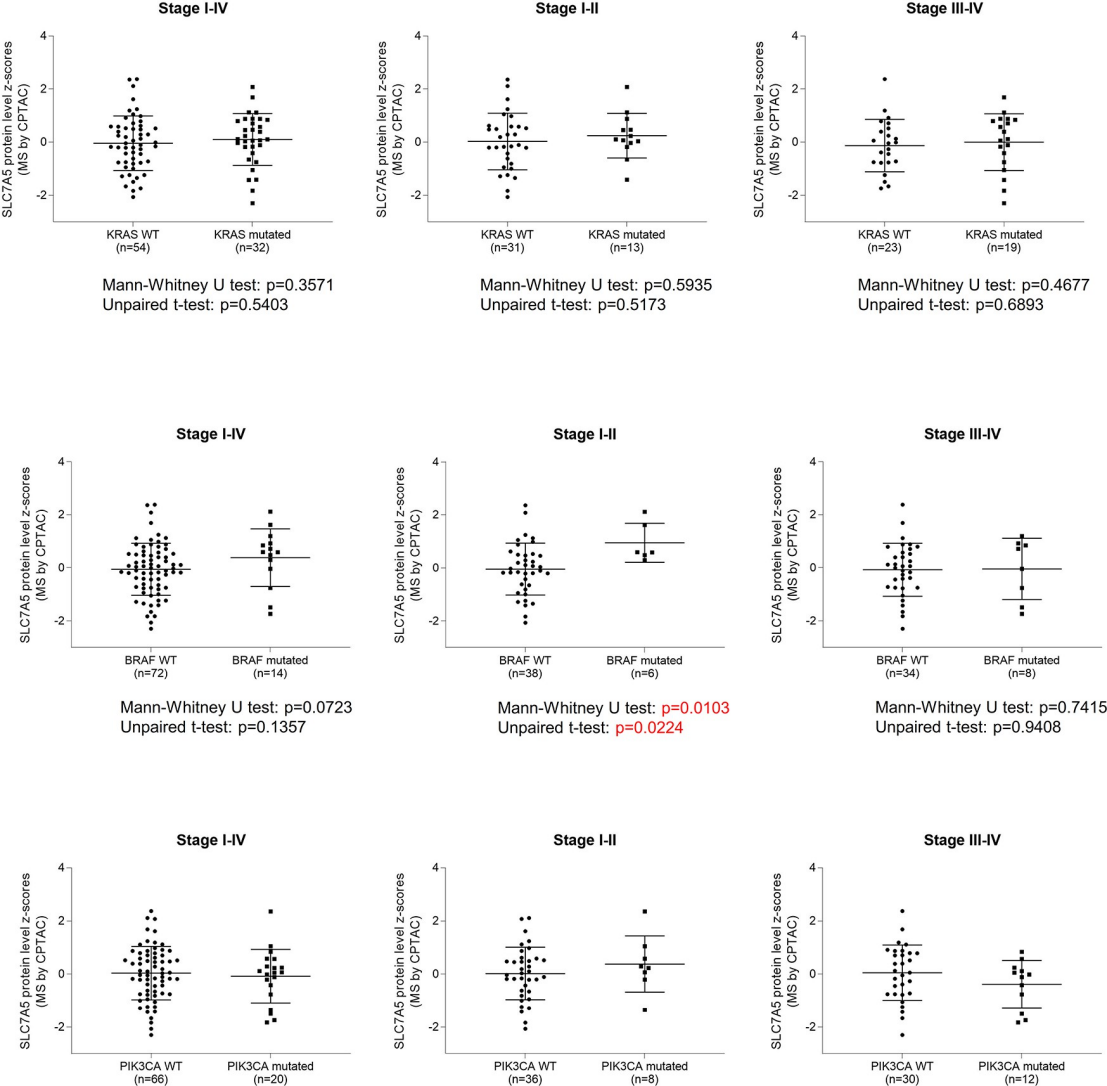
Comparison of SLC7A5 protein expression levels in colorectal cancers with and without *KRAS*, *BRAF*, or *PIK3CA* mutations. The analysis was based on the mass spectrometry protein sequencing data from the CPTAC database. The Y-axis represents SLC7A5 protein level z-scores. SLC7A5 protein expression levels are significantly higher in BRAF-mutated tumors than wild-type tumors.

Since *KRAS*, *BRAF* and *PIK3CA* gene mutations are frequently present in colon cancers, we asked whether these mutations affect SLC7A5 expression in colorectal cancer tissue. According to the protein expression profiles from the CPTAC colorectal cancer cohort, SLC7A5 expression levels has no significant correlations with *KRAS* or *PIK3CA* mutation status in all stages of colorectal cancers. However, while late-stage BRAF-mutated and -wildtype cancers showed no significant difference, early-stage *BRAF*-mutated tumors had significantly higher levels of SLC7A5 protein expression than *BRAF*-wildtype tumors (Fig 3).

Since abundant transcriptomic data is available in the TCGA database, we examined whether SLC7A5 mRNA expression were affected by *KRAS*, *BRAF*, or *PIK3CA* mutation status. Among the 518 cases of colorectal cancer, SLC7A5 mRNA expression levels did not show significant difference between wildtype- and mutated-type colorectal cancers with regard to KRAS, BRAF, or PIK3A mutation (S2 Fig).

**Fig 4:**
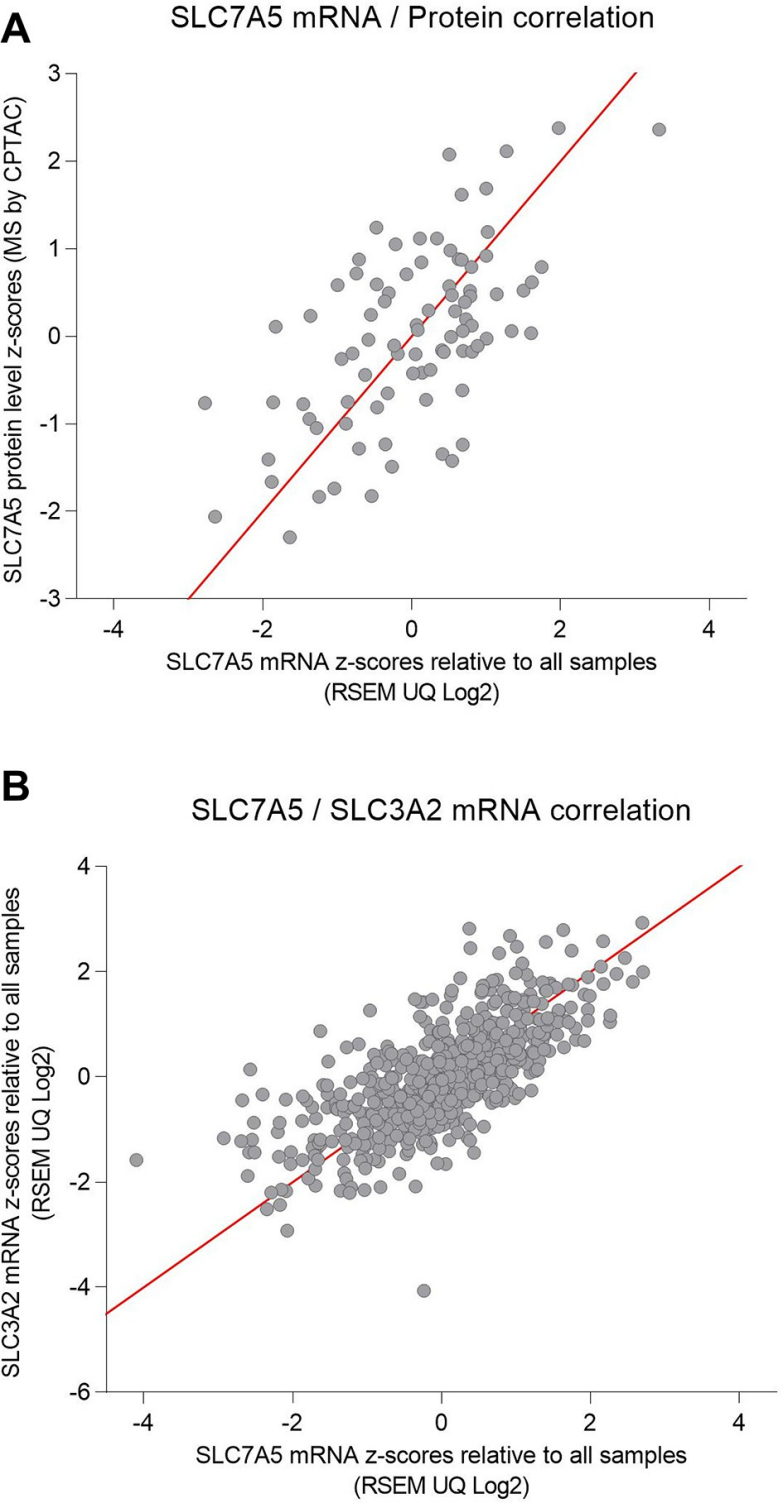
(**A**) The analysis of the SLC7A5 protein and mRNA expressions in the 86-case CPTAC colorectal cohort revealed significant consistency between protein and gene transcription levels. Spearman’s rank correlation coefficient r = 0.5360, p<0.0001. (**B**) SLC7A5 mRNA expression showed the most significant positive correlation with SLC3A2 mRNA expression among all gene transcripts in the 592-case TCGA colorectal cancer cohort. Spearman’s rank correlation coefficient r = 0.7291, p<0.0001.

To verify whether the protein and mRNA expressions of SCL7A5 were consistent in colorectal cancer, we analyzed the gene transcript and protein expression profiles of SLC7A5 in both the TCGA and CPTAC colorectal cancer cohorts. Indeed, SLC7A5 protein and mRNA expression levels were significantly positively correlated (Fig 4A).

### SLC7A5 co-expression network in colorectal cancer

To understand how SLC7A5 expression may be linked to other gene transcripts in colorectal cancer, we analyzed the TCGA colorectal cancer cohort and identified a network of gene transcripts whose expressions were positively correlated with SLC7A5. The top 147 co-expressed genes with Spearman’s correlation coefficient of >0.4 were listed in S2 Table.

Among these co-expressed gene transcripts, SLC3A2 showed the highest correlation (Spearman’s rank correlation coefficient of 0.7314, p = 7.71e-89 and q = 1.53e-84) (Fig 4B). SLC7A5 typically forms a heterodimer with SLC3A2 (CD98 or 4F2 heavy chain) to become a functional transporter [9]. In the SLC7A5/SLC3A2 complex, the catalytic subunit SLC7A5 features a canonical Leu T-fold and exhibits an unusual loop structure on transmembrane helix 6, creating an extended cavity that accommodates bulky amino acids and drugs [10]. This complex promotes tumorigenesis with SLC7A5 importing essential amino acids and promoting mTORC1 activity and SLC3A2 engaging other receptors [32]. Our finding here confirms that SLC7A5 and SLC3A2 were indeed co-expressed and likely form the typical heterodimer in its transporting function in colorectal cancer.

**Fig 5:**
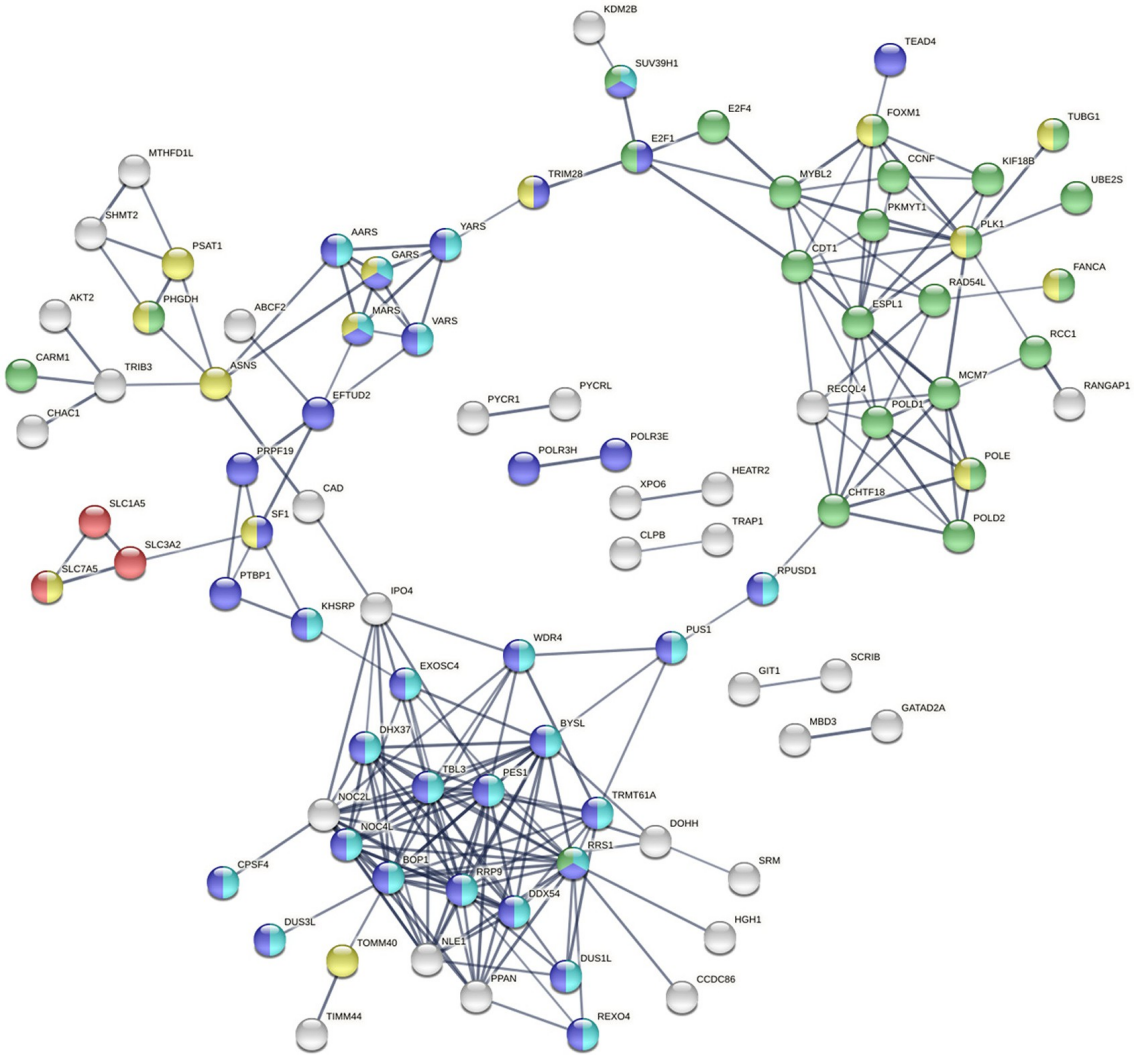
SLC7A5-network showing genes/proteins that were positively co-expressed in the 592-case TGCA colorectal cancer cohort. Only those with Spearman’s rank correlation coefficient >0.4 were included in the network. Only interactions with high confidence (0.7/1.0) are shown in the figure. Amino acid transporters are colored (red). According to Gene Ontology Biological Process annotation, the SLC7A5-network is significantly enriched in ncRNA metabolic process (aqua), RNA metabolic process (blue), and cell cycle (green). Proteins/genes colored yellow are also commonly found in lymphocytes.

To gain functional insight of SLC7A5 in colorectal cancer, we examined the network of the top 147 transcripts that positively co-expressed with SLC7A5 (Fig 5 and S2 Table). Gene Ontology Biological Process (GOBP) analysis by STRING indicated that this SLC7A5-network is significantly enriched for ncRNA metabolic process (29 proteins involved, false discovery rate 1.28e-14), metabolic process of RNA (42 proteins) and tRNA (13 proteins), cell cycle (26 proteins), and mitotic cell cycle process (21 proteins, false discovery rate 5.60e-06).

In line with tRNA processing function, the SCL7A5-network included five tRNA-synthases, including MARS, AARS, GARS, YARS, and VARS and, which specifically conjugate tRNAs with their cognate methionine, alanine, glycine, tyrosine, or valine. We also identified SLC1A5 (ASCT2) as a top co-expresser, which is in line with literature [33, 34]. Interestingly, this network identified POLD1 and POLE, the only two proteins included in the DNA replication proofreading (annotation from Gene Ontology Biological Process). Surprisingly, based on the tissue expression database [35], the SCL7A5-coexpression network identified in colorectal cancer matched best with blood cancer cell (35 proteins, false discovery rate 7.23e-10) and leukemia cell (31 proteins, 5.67e-09). In addition, 18 proteins were matched to lymphocytes (false discovery rate, 0.00058) and 24 proteins were matched to lymphoid tissue (false discovery rate, 0.0277), including SLC7A5 itself.

In this study, we investigated two well-annotated cohorts of colorectal cancer tissues, including a large 575-case early-stage cohort and a 106-case late-stage cohort, and found that SLC7A5 protein expression is a prognostic marker in early-stage colorectal cancer. With 575 cases, our study is the largest cohort study of early-stage colorectal cancer at present time. A high expression of SLC7A5 is significantly correlated with longer overall survival and disease-free survival for early-stage cancer patients. This difference is particularly pronounced in patients with stage II disease. This finding is important because cancer at stage II is critical for risk stratification, and it is when important clinical decision is made as to whether adjuvant therapies should be administered after surgery.

In this study, we detected high expression of SCL7A5 protein in 72% of early-stage and 56.6% of late-stage colorectal cancer cases but none to extremely low expression in normal colonic tissue. The expression patterns are consistent with previous reports, although the prognostic value of SLC7A5 remained controversial thus far [25, 26, 36]. In a mixed cohort, SLC7A5 was absent in colonic mucosa and most benign lesions but began to appear in tubular adenomas and further increased during the adenoma-to-carcinoma transition, although it was not prognostic [25]. SLC7A5 expression was found to be higher in sporadic colorectal tumors than in ulcerative colitis-associated neoplasia, and high expression was unfavorable in phase IV adenocarcinoma [36]. In 147 colorectal cancer patients who underwent surgical resection, SLC7A5 was identified in 80% of the tumor specimens, with positive expression linked to poor diagnosis [2]. In a cohort of 210 colorectal specimens, SLC7A5 high expression was observed in 72.4% of cancer and 30% of colonic adenoma and was associated with venous invasion [26]. In our study, we examined large and well-defined cohorts and found that SLC7A5 expression was prognostic only in early-stage but not late-stage colorectal cancer.

Although SCL7A5 is an important transporter of essential amino acids for cell activities, surprisingly, its functions in colorectal cancer have not been extensively characterized. We therefore analyzed the network of proteins/gene transcripts that were positively co-expressed with SLC7A5 in colorectal cancer. Interestingly, the SCL7A5-network turned out to be significantly associated with ncRNA processing and mitotic cell cycle. Transfer RNAs (tRNAs) are a type of ncRNAs that decode mRNAs in protein synthesis. Indeed, we found several tRNA-aminoacyl synthases among the top SLC7A5 co-expressers. Increased SLC7A5 expression results in increased amino acid uptake into cells, which with increased tRNA synthases will increase protein synthesis that underpins cell proliferation. The SLC7A5-coexpression network analysis thus provides consistent evidence to link SLC7A5 function to amino acid uptake, tRNA/ncRNA processing, and cell cycle.

SLC7A5 is a well-known marker of activated lymphocytes such as NK cells, T cells and B cells [37–41]. SLC7A5 may act as a double-edged sword in cancer development, while its overexpression in cancer cells promote tumor growth, its overexpression in lymphocytes could boost immune responses to cancer. Cancer with increased SLC7A5 expression may be accompanied by increased immune reactions. For example, in triple negative HER2+ and luminal B tumors, SLC7A5/SLC3A2 expressions were variably associated with PDL1/PD1+, CD68+, and CD20+ immune cell infiltrates, which could either support or counter tumor progression [42]. NK cells integrate signals received from tumor interactions and IL2 to induce robust increases in SLC7A5 expression, mTORC1 and cMyc metabolic signaling pathways, and prolonged antitumor response [38]. In T cells, the intracellular supply of large neutral amino acids was regulated by pathogens and TCR, and SLC7C5 was the single transporter that mediated uptake of large neutral amino acids in activated T cells [40, 41]. In B cells, leucine influx through SLC7A5 was found to critically regulate mTORC1 activity and the immunological responses [39].

## Conclusions

In summary, using large well-annotated cohorts of colorectal cancer tissue, this study identified increased tissue protein expression of SLC7A5 to be a favorable prognostic marker in early-stage colorectal cancer. Together with data from TCGA and CPTA, our study also identified a SLC7A5 co-expression network that links SLC7A5 function to ncRNA processing and cell cycle in colorectal cancer. Our findings provide new insights for further exploring SLC5A as a promising marker for cancer diagnosis and risk stratification as well as a target for therapy development against deadly colorectal cancer at its early stage.

## Supporting information

S1 Fig Overall survival (OS) or disease-free survival (DFS) of stage II colorectal cancer patients stratified by SLC7A5 protein expression. High express correlated significantly with better clinical outcome. High expression refers to cancer tissue with H-score ≥40 for SLC7A5 protein. MSS, microsatellite stability; MSI, microsatellite instability.
(TIF)

S2 Fig *SLC7A5* mRNA expression in the colorectal adenocarcinoma TCGA cohort as a function of wild type (WT) vs. mutated genomic status of *KRAS*, *BRAF*, or *PIK3CA*. *SLC7A5* mRNA expression did not appear to be affected by these gene mutations.
(TIF)

S1 Table Univariate and multivariate analyses of patient survival in the MSS subtype of early-stage colorectal cancer. (DOCX)

S2 Table *SLC7A5* co-expression analysis. (XLSX)
