# Supplementary material for: Protein expression of the amino acid transporter SLC7A5 in tumor tissue is prognostic in early-stage colorectal cancer
Source: PLoS One. 2024 May 9;19(5):e0298362. doi: 10.1371/journal.pone.0298362 (PMC11081336; doi:10.1371/journal.pone.0298362)
Supplement: S1 Table — (DOCX) [file pone.0298362.s003.docx]

**S1 Table.** Univariate and multivariate analyses of patient survival in the MSS subtype of early-stage colorectal cancer

|  | **Overall survival** | | | | **Disease-free survival** | | | |
| --- | --- | --- | --- | --- | --- | --- | --- | --- |
| Variable | Univariate | | Multivariate | | Univariate | | Multivariate | |
|  | HR (95% CI) | p-value | HR (95% CI) | p-value | HR (95% CI) | p-value | HR (95% CI) | p-value |
| **Gender** | 0.95 | 0.5642 |  |  | 0.87 | 0.1188 |  |  |
| (male vs. female) | (0.79-1.13) |  |  |  | (0.74-1.03) |  |  |  |
| **Age (years)** | 0.69 | <0.0001 | 0.67 | <0.0001 | 0.76 | 0.0010 | 0.74 | 0.0006 |
| (>70 vs. ≤70) | (0.57-0.82) |  | (0.55-0.80) |  | (0.64-0.89) |  | (0.63-0.88) |  |
| **Tumor location** | 0.87 | 0.1396 |  |  | 0.92 | 0.3034 |  |  |
| (right vs. left) | (0.73-1.05) |  |  |  | (0.78-1.08) |  |  |  |
| **Histology** | 1.08 | 0.6873 |  |  | 0.99 | 0.9334 |  |  |
| (mucinous vs. other) | (0.77-1.66) |  |  |  | (0.72-1.43) |  |  |  |
| **Tumor differentiation** | 0.72 | 0.2396 |  |  | 0.78 | 0.3727 |  |  |
| (G3 vs. G1/2) | (0.47-1.30) |  |  |  | (0.51-1.41) |  |  |  |
| **Lymphovascular invasion** | 0.67 | 0.0028 | 0.70 | 0.0095 | 0.61 | <0.0001 | 0.64 | 0.0003 |
|  | (0.53-0.86) |  | (0.55-0.91) |  | (0.50-0.76) |  | (0.52-0.81) |  |
| **Perineural invasion** | 0.55 | 0.0039 | 0.63 | 0.0212 | 0.61 | 0.0116 | 0.73 | 0.1067 |
|  | (0.40-0.81) |  | (0.45-0.93) |  | (0.44-0.88) |  | (0.53-1.08) |  |
| **AJCC stage** | 0.74 | 0.0019 | 0.80 | 0.0262 | 0.72 | 0.0004 | 0.78 | 0.0070 |
| (II vs. I) | (0.60-0.90) |  | (0.65-0.97) |  | (0.60-0.87) |  | (0.64-0.93) |  |
| **SLC7A5 expression** | 1.23 | 0.0441 | 1.18 | 0.0936 | 1.21 | 0.0390 | 1.16 | 0.1112 |
| (low vs. high) | (1.01-1.48) |  | (0.97-1.43) |  | (1.01-1.44) |  | (0.97-1.38) |  |
